# Effect of ‘hand and foot acupuncture with twelve needles’ on hemiplegia patients with ‘qi deficiency and blood stasis’ syndrome in the convalescent stage of Ischaemic stroke: study protocol for a randomised controlled trial

**DOI:** 10.1186/s13063-021-05128-5

**Published:** 2021-03-18

**Authors:** Wei-Hao Fang, Gui-Ling Wang, Qiang Liu, Xiao Ding, Zhen-Yao Wang, Xin-Wei Wang, Xiao-Wei Yang, Yang Yang, Da-Wei Zhang, Qing Wei, Hu Zhang

**Affiliations:** 1 Shunyi Hospital, Beijing Traditional Chinese Medicine Hospital, Beijing, 101300 China; 2 Beijing Hospital of Traditional Chinese Medicine, Beijing, 100010 China; 3 World Federation of Chinese Medicine Societies, Beijing, 100101 China

**Keywords:** Ischaemic stroke, Hemiplegia, Acupuncture, Randomised controlled trial, Study protocol

## Abstract

**Background:**

Hemiplegia is a common sequela after stroke, and acupuncture is one of the most common physical therapies used to treat hemiplegia during the recovery stage after ischaemic stroke. ‘Hand and foot acupuncture with twelve needles’ is an acupuncture treatment performed after stroke. The principal objective of this study is to assess the efficacy and safety of ‘hand and foot acupuncture with twelve needles’ for hemiplegia in the convalescent stage of ischaemic stroke.

**Methods:**

This is the protocol for a randomised, controlled clinical trial with two groups: a ‘hand and foot acupuncture with twelve needles’ group and a routine acupuncture group. A total of 208 participants will be randomly assigned to two different groups in a 1:1 ratio and will undergo conventional rehabilitation. Limb function will be evaluated by the simplified Fugl-Meyer assessment scale, Barthel Index, modified Ashworth scale and National Institute of Health stroke scale. The participants will be evaluated at baseline (on the day of enrolment) and followed up at 2 weeks, 1 month, 2 months and 3 months after enrolment.

**Discussion:**

The results of this study will provide evidence on the effectiveness of ‘hand and foot acupuncture with twelve needles’ in the treatment of limb dysfunction that can be used for future evaluations.

**Trial registration:**

Chictr.org.cnChiCTR1900021774. Registered on 8 March 2019

## Administrative information

The order of the items has been modified to group similar items (see http://www.equator-network.org/reporting-guidelines/spirit-2013-statement-defining-standard-protocol-items-for-clinical-trials/).

**Table:** 

Title{1}	Effect of “Hand and Foot Acupuncture with Twelve Needles” on Hemiplegia Patients with “Qi Deficiency and Blood Stasis” Syndrome in the Convalescent Stage of Ischemic Stroke: Study Protocol for a Randomized Controlled Trial
Trial registration {2a and 2b}.	Chictr.org.cn, ChiCTR1900021774. Registered on 8 March 2019.
Protocol version {3}	Protocol version 2.0, dated August, 2018.
Funding {4}	The trial is funded by the Capital Special Foundation of Clinical Application, Beijing Municipal Science and Technology Commission, China (Z171100001017150).
Author details {5a}	Wei-Hao Fang ^1^ (e-mail: zyfwh85@163.com), Gui-Ling Wang ^2^ (e-mail: wangguiling100@163.com), Qiang Liu^3^ (e-mail: liuqcn@126.com), Xiao Ding^1^ (e-mail: 34221457@qq.com), Zhen-Yao Wang ^1^ (e-mail: wangzhenyao8@126.com), Xin-Wei Wang ^1^ (e-mail: 283511767@qq.com), Xiao-Wei Yang ^1^ (e-mail: 27325795@qq.com), Yang Yang^1^ (e-mail: aeon-ren@sohu.com), Da-Wei Zhang ^1^(david989898@163.com), Qing Wei^1^ (e-mail: 1961450463@qq.com), Hu Zhang^1*^ (e-mail: zhanghu0709@sohu.com) 1 Shunyi Hospital, Beijing Traditional Chinese Medicine Hospital, Beijing, 101300, China, 2 Beijing Hospital of Traditional Chinese Medicine, Beijing, 100010, China, 3 World Federation of Chinese Medicine Societies, Beijing, 100101, China.
Name and contact information for the trial sponsor {5b}	Hu Zhang, e-mail: syzyykfk@163.com, Shunyi Hospital, Beijing Traditional Chinese Medicine Hospital, Beijing, China.
Role of sponsor {5c}	The sponsor participated in the design of this study and will be involved in the interpretation of the results. The funder provided research funding but had no role in the design and will not be involved in the conduct of the trial.

## Introduction

### Background and rationale {6a}

In recent years, the incidence of stroke and the associated mortality rate in China have increased [1]. Epidemiological findings show that the age-standardised prevalence, incidence and mortality rates were 1114.8 per 100,000, 246.8 per 100,000 and 114.8 per 100,000, respectively, in 2013 [2]. Ischaemic stroke accounts for 69.6–77.8% of all strokes [2]. China ranks first amongst all countries in terms of the DALYs (disability-adjusted life years) resulting from stroke [1]. Stroke has become the most common and major cause of disability [3, 4]. Approximately 70–80% of the existing stroke patients have lost, to some extent, their ability to work [5]. Hemiplegia, one of the most common sequelae after stroke, directly affects the quality of work and life of patients and imposes a heavy economic burden on society and families [6].

Evidence-based medicine (EBM) has confirmed that rehabilitation training is the most effective treatment for decreasing the disability rate [7, 8]. In modern medicine, the conventional treatments also include exercise therapy [9], traditional Chinese medicine (TCM) exercise therapy [10, 11], medicine [12], acupuncture [12, 13], transcranial magnetic stimulation (TMS) [14–16], mirror therapy [17] and music-supported therapy [18]. In China, acupuncture is one of the most common physical therapies performed to treat stroke-associated hemiplegia and can reduce muscle tension in patients with spastic hemiplegia after stroke [19, 20]. Acupuncture is known to yield better results when the intervention is implemented earlier [21]. However, due to the complexity of patients’ conditions, the processes for selecting safe and effective acupuncture points as well as performing manipulation according to the actual patient conditions need to be explored further.

‘Hand and foot acupuncture with twelve needles’ has been widely used to treat various diseases, such as multiple sclerosis [22] and insomnia [23] and is commonly used for stroke patients. In clinical practice, ‘hand and foot acupuncture with twelve needles’ is often used to treat apoplexy [24]; however, studies such as clinical trials supporting this contention are relatively scarce. Some studies have shown that ‘hand and foot acupuncture with twelve needles’ can effectively reduce the lesion excitability of the contralateral primary motor cortex and improve the excitability imbalance in the bilateral cortex in patients with ischaemic stroke, and there is a significant correlation between the decline in excitability in the cortex and clinical efficacy [25]. Therefore, we designed a clinical trial to observe the primary effects of ‘hand and foot acupuncture with twelve needles’ on stroke hemiplegia.

### Objectives {7}

The objective of this study is to compare the effectiveness of ‘hand and foot acupuncture with twelve needles’ with that of routine acupuncture technique in hemiplegia patients with ‘qi deficiency and blood stasis’ syndrome in the convalescent stage of ischaemic stroke. This study also aims to assess the difference in the effects of these two techniques on motor function, independence in activities of daily living (ADLs), limb spasticity and neurological deficits.

### Trial design {8}

**Fig. 1:**
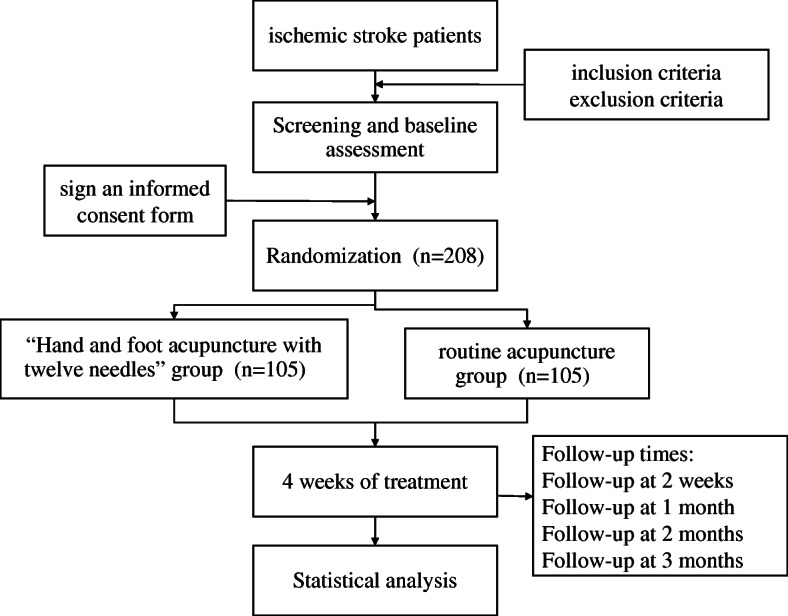
Trial flow chart

**Fig. 2:**
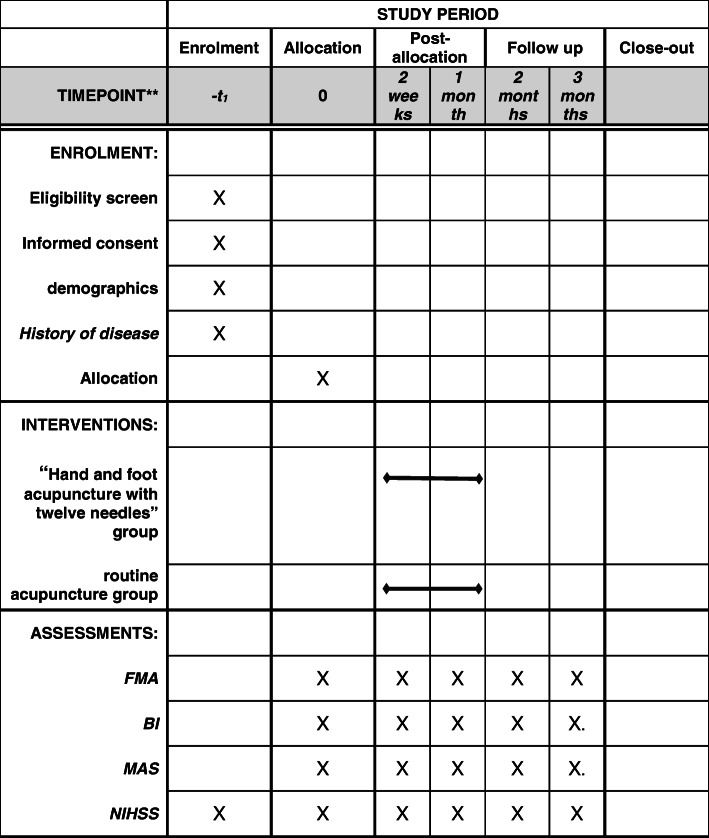
SPIRIT figure

This is an open-label, single-centre, parallel design, 2-arm (allocation ratio 1:1), superiority, randomised controlled trial. A flow diagram of this study protocol is shown in Fig. 1, and the treatment and outcome measures are presented in Fig. 2.

## Methods: participants, interventions and outcomes

### Study setting {9}

Stroke patients with hemiplegia will be recruited from a rehabilitation clinic or ward of Shunyi Hospital, Beijing Traditional Chinese Medicine Hospital in Beijing, China.

### Eligibility criteria {10}

#### Inclusion criteria

Patients satisfying the following six conditions will be included:

A. Diagnosis of ‘apoplexy’ according to TCM and cerebral infarction confirmed by computed tomography or magnetic resonance imaging
B. Age of 35 to 80 years
C. A duration from stroke onset of 2 weeks to 3 months
D. National Institute of Health stroke scale (NIHSS) score of 5–22
E. A limb strength score below grade 4 and limb dysfunction
F. Qi deficiency syndrome and blood stasis syndrome confirmed by the ‘Ischemic Ischaemic Stroke TCM Syndrome Diagnostic Scale (ISTCMDS)’

#### Exclusion criteria

Patients with any of the following conditions will be excluded:

A. Intracerebral haemorrhage or subarachnoid haemorrhage confirmed by computed tomography or magnetic resonance imaging
B. Recurrent stroke with a neurofunctional disability that can affect the results of this study
C. A history of a brain tumour, traumatic brain injury, haematopathy, etc.
D. Other severe diseases that can affect cerebral infarction therapy outcomes and mental impairment
E. A pregnant or lactating status
F. Participation in another clinical trial for a drug, acupuncture or rehabilitation
G. Skin lesions and other skin diseases at acupuncture points

### Who will take informed consent? {26a}

Consent will be obtained by one of the researchers in the presence of a witness. After providing written informed consent, eligible patients will be asked to complete a general information form including their name, sex, age and medical history and complete the same day scale as the baseline assessment. A researcher who is a neurologist with more than 5 years of experience will evaluate the NIHSS, Simplified Fugl-Meyer assessment scale (FMA), modified Ashworth scale (MAS) and Barthel Index (BI) scores for each participant. These scores will be used to determine the eligibility of a patient for the study in accordance with the inclusion and exclusion criteria. The personal information of potential as well as enrolled participants will be collected and stored confidentially.

### Additional consent provisions for collection and use of participant data and biological specimens {26b}

On the consent form, the participants will be asked whether they permit the continued use of their data in case of withdrawal from the trial. The participants will also be asked for permission to share relevant data with the research team from the universities taking part in the study or with regulatory authorities, when relevant. This trial does not require the collection of biological specimens for storage.

## Interventions

### Explanation for the choice of comparators {6b}

In China, stroke patients are often treated with acupuncture. Thus, the patients can easily determine whether fake acupuncture is used. Therefore, we decided to perform standard procedure acupuncture rather than sham acupuncture for the control group.

### Intervention description {11a}

#### ‘Hand and foot acupuncture with twelve needles’ group

1. ‘Hand and foot acupuncture with twelve needles’ involves 12 acupoints, including hegu (LI4, bilateral), neiguan (PC6, bilateral), quchi (LI11, bilateral), zusanli (ST36, bilateral), yanglingquan (GB34, bilateral) and sanyinjiao (SP6, bilateral).
2. The patient will be asked to rest in a supine position, and the skin at the acupoints will be disinfected routinely. Disposable acupuncture needles will be inserted into the abovementioned acupoints (15–20 mm deep). After twirling, these needles will be left in place for 20 min. The procedure will be repeated five times per week for 4 weeks.

### Routine acupuncture group

(3) Routine acupuncture [26] will be performed in accordance with the Evidence-based Guidelines of Clinical Practice in Chinese Medicine Internal Medicine, and needles will be placed at neiguan (PC6), shuigou (DU26), sanyinjiao (SP6), jiquan (HT1), chize (LU5), weizhong (BL40), jianyu (LI15), shousanli (LI10), hegu (LI4), huantiao (GB30), yanglingquan (GB34), xuanzhong (GB39) and taichong (LR3) on the hemiplegic side.
(4) The patient will be asked to rest in a supine position, and the skin at the acupoints will be disinfected routinely. Disposable acupuncture needles will be inserted into the above acupoints at a depth of 15–20 mm, except at huantiao where the needle will be inserted 30 mm deep. After twirling, these needles will be left in place for 20 min. The procedure will be repeated five times per week for 4 weeks.

### Criteria for discontinuing or modifying allocated interventions {11b}

There will be no special criteria for discontinuing or modifying the allocated interventions.

### Strategies to improve adherence to interventions {11c}

Patients will be followed up via telephone calls or by outpatient follow-up systems. We also plan to hold awareness meetups regularly to encourage patient involvement. All assessments will be performed at baseline (on the day of enrolment) and 2 weeks, 1 month, 2 months and 3 months after enrolment (follow-ups).

### Relevant concomitant care permitted or prohibited during the trial {11d}

#### Routine interventions

1. Symptomatic treatment according to the 2010 Guidelines of Chinese Stroke Prevention and Control includes the prevention and treatment of complications, glycaemic control and blood pressure control.
2. Rehabilitation training includes physical therapy (PT) and occupational therapy (OT) for 5 days per week.
3. Acupuncture will be performed in accordance with the Standards for Reporting Interventions in Clinical Trials of Acupuncture (STRICTA) guidelines, after the participant’s condition is stabilised by TCM practitioners (trained medical acupuncturists) using routinely disinfected disposable acupuncture needles (size 0.30 × 40 mm, Suzhou Dongbang Medical Apparatus Co., Ltd., Suzhou, China).

### Provisions for post-trial care {30}

There is no anticipated harm associated with or compensation for trial participation.

### Outcomes {12}

#### Primary outcome measure

The simplified FMA [27] will be used to estimate the capacity of autonomous movement of the limb and includes items on reflex activity, flexor synergy, extensor synergy, wrist and hand, coordination and speed. The total score possible is 100 points, and motor function of the upper limbs (33 items, 66 points) and lower limbs (17 items, 34 points) will be assessed.

#### Secondary outcome measures

The NIHSS [28] will be used to assess the neurological deficits related to stroke and includes items on consciousness, language, movement, sensation, ataxia, eye movement, visual field and others. The scores range from 0 to 42, with higher scores indicating more severe neurological deficit.

The MAS [29] is a grading standard used to measure muscle tone with a scale ranging from 0 to 4 points.

The BI [30] will be used to determine the functional status of patients’ daily activities, which includes bowel control, feeding, dressing, access to the toilet, grooming, bathing, climbing from a wheelchair to bed and back to a wheelchair, walking 45 m on a horizontal surface and going up and down the stairs. The scores range from 0 to 100.

### Participant timeline {13}

All patients will be assessed by a researcher using the above scales on the day of enrolment and 2 weeks, 1 month, 2 months and 3 months after enrolment.

### Sample size {14}

The primary efficacy parameter is the change in the FMA score from baseline to 3 months after the treatment [31]. According to our preliminary test and a previous study, the primary efficacy parameter (FMA score) of the control group (rehabilitation treatment) will increase by 12.11 points and that of the treatment group (scalp acupuncture combination rehabilitation treatment) will increase by 25.82 points. The FMA average standard deviation is expected to be approximately 4. Considering a one-sided 5% significance level, 80% power and the above data (*μ*_1_ = 25.82, *μ*_2_ = 12.11, *σ* = 4, *α* = 0.025, 1–*β* = 0.8), according to NCSS-PASS V20.0.3 software (https://www.ncss.com/software/pass/), approximately 86 participants will be required in each group to achieve a sufficient sample size. Accounting for a dropout rate of 20%, each group must include 104 initial participants. Therefore, we plan to include a total of 208 participants divided equally into two groups.

### Recruitment {15}

Trial participants with stroke are being recruited by clinicians from outpatient clinics at Shunyi Hospital, Beijing Traditional Chinese Medicine Hospital. Moreover, information flyers containing the details of the trial are being posted at outpatient clinics of respective institutions for greater exposure. The researchers will enrol patients in accordance with the inclusion and exclusion criteria on the day of admission. Patient recruitment started in January 2021.

## Assignment of interventions: allocation

### Sequence generation {16a}

Commonly used factors for patient stratification include age, sex, body mass index (BMI), smoking habits, and alcohol consumption habits. In this study, the patients are expected to have a large age gap. Therefore, we have chosen age as the blocking factor. All participants will be randomly assigned to one of two groups (‘hand and foot acupuncture with twelve needles’ group and routine acupuncture group) in a 1:1 ratio. The generation and allocation of a random sequence for group assignment will be conducted by the World Federation of Chinese Medicine Societies in China. A block randomisation method (with a block size of four) will be used to generate the random allocation sequence; opaque sealed envelopes containing the predetermined computer-generated numbers will be used to ensure allocation concealment.

### Concealment mechanism {16b}

Random numbers will be placed in sequentially numbered, opaque, sealed envelopes.

### Implementation {16c}

A doctor who is not involved in this study will store the envelopes with random numbers. The researcher who registers the participants will open the envelopes and assign the participants to the intervention groups.

## Assignment of interventions: blinding

### Who will be blinded {17a}

In this study, the outcome assessors and data analysts will be blinded, but the participants and researchers will not be blinded.

### Procedure for unblinding if needed {17b}

The design is open-label with only the outcome assessors and data analysts being blinded so unblinding will not occur.

## Data collection and management

### Plans for assessment and collection of outcomes {18a}

All researchers, including the acupuncturists, outcome assessors, data collectors, data managers, data entry personnel and statisticians, will undergo training before performing standard procedures and data management. A detailed instruction manual will be prepared to ensure consistency and standardisation of data collection and assessment. All data of the participants will be recorded in case report forms (CRFs) by a data collector during the recruitment period and upon the completion of the treatment and follow-up phases. Physicians trained to administer the assessment tools required for this study will be responsible for collecting these data to ensure data are accurate and thoroughness. The physicians will complete the general information form during the assessment. The outcomes will be assessed by a professional from the World Federation of Chinese Medicine Societies.

### Plans to promote participant retention and complete follow-up {18b}

We will follow up the patients for 2 months, either by readmission or outpatient medical consultation. We also plan to hold events to spread awareness about the treatments, which will help ensure patient compliance.

### Data management {19}

All collected data will be entered into the electronic data capture system (EDC, Goodwill Information Technology Co, Ltd., Beijing, China) by trained researchers. To ensure the accuracy of the data, we will randomly check the entered data against the recorded information.

### Confidentiality {27}

The personal data of potential study volunteers as well as the participants will be kept strictly confidential. The participants will be identified by their initials rather than their names. All files will be stored in locked file cabinets which only the researchers will have access to. When the research outcomes are compiled in a publication, no personal information will be disclosed.

### Plans for collection, laboratory evaluation and storage of biological specimens for genetic or molecular analysis in this trial/future use {33}

This trial does not require the collection of biological specimens for storage.

## Statistical methods

### Statistical methods for primary and secondary outcomes {20a}

All analyses will be carried out using IBM SPSS Statistics for Windows, version 22.0 (IBM Corp., Armonk, NY, USA). The measurement data will be expressed as the mean ± standard deviation values. For the outcome measures, the changes in each score and differences between the two groups will be evaluated. Normally distributed data will be analysed using a *t* test, and the non-normally distributed data will be analysed by a rank-sum test. An independent *t* test or rank-sum test will be performed to assess the differences between the two groups. In addition, the count data will be expressed by frequency and analysed by a chi-square test.

### Interim analyses {21b}

Descriptive statistics will be used to describe the general status characteristics of patients in detail at baseline, such as gender, age, disease course, and stroke risk factors. Mann-Whitney U test will be used to analyse value changes of FMA, NIHSS, MAS, and BI scores among the groups. Repeated measures analysis of variance (ANOVA) will be used to analyse value changes of FMA, NIHSS, MAS, and BI scores across five testing time points (weeks 0, 2, 4, 8, and 12). Adverse events will be summarised with descriptive statistics.

### Methods for additional analyses (e.g. subgroup analyses) {20b}

Subgroup analysis will be conducted according to the severity of impairment in the limbs and age (e.g. NIHSS 5–15, NIHSS 15–20, NIHSS 20–22; age 35–45, age 45–65, age 65–80, respectively).

### Methods in analysis to handle protocol non-adherence and any statistical methods to handle missing data {20c}

If data are missing, the missing data field will be populated with the value of the last measurement.

### Plans to give access to the full protocol, participant level-data and statistical code {31c}

The datasets analysed during the current study will be available from the corresponding author upon reasonable request.

## Oversight and monitoring

### Composition of the coordinating centre and trial steering committee {5d}

The World Federation of Chinese Medicine Societies will manage the statistical analysis. The endpoint adjudication committee is the Ethics Committee of Shunyi Hospital of Traditional Chinese Medicine. The Office of Scientific Research in our hospital is the Trial Steering Committee and will supervise the trial, and this group will meet twice per year.

### Composition of the data monitoring committee, its role and reporting structure {21a}

The data monitoring committee (DMC) was established before the study, is independent of the sponsor and has no competing interests. The committee trains researchers on data management. DMC ensures and monitors the quality and completeness of the recorded data.

### Adverse event reporting and harms {22}

All adverse events will be recorded truthfully in the trial. Regarding safety, the patient’s vital signs will be measured, and physical examinations will be performed. The vital sign measurements will include sitting blood pressure, heart rate, respiration rate and body temperature. For acupuncture, the common and expected adverse events include local haematomas, needle breakage, needle retention after treatment, fainting, unbearable prickling, severe pain, persistent discomfort for more than 1 h after acupuncture, local infections and abscesses. The participants will be instructed to report any abnormal reactions or uncomfortable feelings experienced to any researcher. All unexpected recurrent strokes will be recorded in detail in the CRFs, including the time of occurrence, degree of stroke and plausible causes. Participants with mild and moderate stroke (NIHSS score < 22) will be treated for their symptoms and closely monitored as necessary by the researcher. Severe stroke (NIHSS score ≥ 22) will be reported to the research ethics committee, which will provide medical advice to the research team within 48 h, and the research ethics committee will determine whether the trial should be terminated.

### Frequency and plans for auditing trial conduct {23}

The World Federation of Chinese Medicine Societies will manage the statistical analysis. The endpoint adjudication committee is the Ethics Committee of Shunyi Hospital of Traditional Chinese Medicine. The Office of Scientific Research in our hospital is the Trial Steering Committee and will supervise the trial, and the committee will meet twice per year.

### Plans for communicating important protocol amendments to relevant parties (e.g. trial participants, ethical committees) {25}

We will first notify the sponsor and funder of any changes made to the protocol. Then, the PI will notify the centres, and a copy of the revised protocol will be sent to the PI to be added to the investigator site file. We will state all deviations from the previous protocol, which will be fully documented using a breach report form. We will update the protocol in the clinical trial registry.

### Dissemination plans {31a}

The results will be published in a paper after the completion of the study.

## Discussion

According to the results of previous studies [32], acupuncture is beneficial for motor function recovery in stroke patients with hemiplegia and is considered to be safe [33, 34]. The mechanism of motor recovery due to acupuncture in stroke hemiplegia may be related to brain plasticity [35]. Acupuncture in one limb can stimulate bilateral brain areas, increase cerebral blood flow and regulate motor areas damaged by stroke [36]. For example, acupuncture in yanglingquan can regulate multiple brain networks in stroke patients [35]. Thus, it promotes the recovery of hemiplegia limbs.

In this study, ‘hand and foot acupuncture with twelve needles’ includes the acupoints hegu (bilateral), neiguan (bilateral), quchi (bilateral), zusanli (bilateral), yanglingquan (bilateral) and sanyinjiao (bilateral); according to TCM theory (five SHU points of the hands and feet), acupuncture at these points can pass through the meridians, regulating qi and blood.

The FMA can be used to assess motor performance and movement quality in hemiplegic stroke patients [37]. The NIHSS is commonly used to evaluate clinical stroke severity. The MAS is significantly sensitive for assessing changes in the muscle tone of stroke patients with hemiplegia [38]. The BI is highly sensitive to changes in mild stroke patients’ independence performing ADL in the recovery period [39]. Therefore, this study will use the above assessment tools to assess motor function, muscle tension, neurological deficits and patients’ ability to perform ADLs in patients with hemiplegia after stroke.

We present the protocol for a randomised, controlled clinical trial to evaluate the effects of ‘hand and foot acupuncture with twelve needles’. In this study, we will evaluate the effects of different acupuncture treatments on limb dysfunction, limb spasm and patients’ independence in performing ADLs after stroke. The participants will be enrolled for 2 months and followed up for 1 month after the intervention. The data analysts will be blinded to this study design to reduce bias and ensure the quality of this trial. The main limitation of this study is its non-double-blind design. Furthermore, this study will not include data from long-term follow-ups.

### Trial status

This protocol version number is 2.0, dated August 2018. Participant recruitment began on March 8, 2019, and will end on approximately February 28, 2022.

## Abbreviations

DALYs: Disability-adjusted life years
EBM: Evidence-based medicine
TMS: Transcranial magnetic stimulation
ADLs: Activities of daily living
FMA: Fugl-Meyer assessment
BI: Barthel Index
MAS: Modified Ashworth scale
NIHSS: National Institutes of Health stroke scale
ISTCMDS: Ischaemic Stroke TCM Syndrome Diagnostic Scale
PT: Physical therapy
OT: Occupational therapy
STRICTA: Standards for Reporting Interventions in Clinical Trials of Acupuncture
TCM: Traditional Chinese medicine
